# Status of Telemonitoring Services in Diabetes Care in Germany: A Narrative Review

**DOI:** 10.1089/tmr.2020.0012

**Published:** 2021-01-08

**Authors:** Martina Verfürth

**Affiliations:** ^1^ Escuela Internacional de Doctorado UCAM (EIDUCAM) [International Doctoral School], UCAM Universidad Católica San Antonio de Murcia, Guadalupe de Maciascoque, Murcia, Spain.; ^2^ FOM Hochschule für Oekonomie and Management Essen, Essen, Germany.

**Keywords:** chronic care, implementation research, telemedicine

## Abstract

**Background:**

Diabetes mellitus (DM) as a chronic disease is a great medical, organizational, and financial burden on the German healthcare system, and it has risen in epidemiological importance. To ensure healthcare against the background of rising prevalence rates and to reduce costs, it makes sense to supplement treatment of diabetes patients with telemedical services.

**Objectives:**

The aim was to evaluate telemonitoring services for DM patients in Germany and the political/legal environment.

**Materials and Methods:**

A narrative review was conducted to provide a comprehensive and critical analysis of the current knowledge on interactive telemonitoring offerings and influencing factors. A total of 19 publications were considered as relevant in the screening process, and were included in the content analysis.

**Results:**

The results can be differentiated in terms of political/legal requirements, needs, and supply-related aspects. Only four studies focused on the individual care aspects of telemedical care of DM patients.

**Conclusion:**

Telemonitoring measures for chronic diseases in general and for DM in particular have hardly been implemented in Germany so far. Based on the deficiencies and research gaps described earlier, some recommendations can be made. There is a need to set up structure for more interactivity, to expand technical infrastructure, and to close legal gaps. More research focusing on clinical effectiveness is necessary.

## Background

### Diabetes mellitus: burden of the disease

Diabetes mellitus (DM) describes a group of metabolic disorders of the carbohydrate metabolism that are based on (absolute or relative) lack of insulin and that lead to chronic hyperglycemia. The characteristics of this complex disorder include hyperglycemia, glucosuria, acidosis, and ketosis, whereas the leading symptom is a high blood sugar level over a prolonged period of time.^1,2^ DM is divided in different types. Type 1 diabetes mellitus (T1DM) is a heritable autoimmune phenomenon, but environmental factors also play an important role. Type 2 diabetes mellitus (T2DM) is the result of lifestyle factors such as overweight, malnutrition, lack of movement, and consumption of nicotine and alcohol. Type 3 diabetes mellitus (T3DM) includes all rare forms of diabetes. It is caused by surgery or medication and diseases of the adrenal gland or thyroid gland, as well as monogenetic disorders. The fourth and a special type of diabetes is gestational diabetes. In this case, women have increased blood sugar levels during pregnancy because of hormonal changes.^1–3^ Of particular epidemiological importance is T2DM, which is considered a chronic disease and whose prevalence has risen rapidly in industrialized countries in recent decades.^4^ Worldwide, the number of people being treated for DM (especially T2DM) has more than tripled in the past 20 years, from 150 million cases (in 2000) to 460 million cases (in 2019).^5^ In Europe alone, ∼56 million people live with diabetes, which represents 8.5% (and rising) of the population; >90% of patients have T2DM.^4,5^

### Diabetes mellitus in Germany

In Germany, the number of people with diabetes has increased from 0.6 million in the 1960s to 6 million in 2014. In 2017, there were 7.5 million DM patients in Germany, and there are ∼500,000 new diabetes diagnoses every year. In 2019, the Diabetes Atlas of the International Diabetes Federation stated that 9.5 million German people have diabetes. Compared with other European countries, Germany currently has the highest rate of DM patients. It is estimated that there will be 10.1 million people with diabetes in Germany by 2030.^4^ Most of these patients—∼95%—have T2DM. Experts have estimated that there are between 2 and 4.5 million unrecorded cases of diabetes in Germany. This estimate means that >10% of the German population likely has diabetes. There are differences in diabetes prevalence throughout Germany: in eastern Germany it is estimated to be 11.6%, which is substantially higher than the estimated 2.7% in western Germany. People living in cities have a much higher risk (40%) of developing diabetes compared with people living in rural areas.^5–10^

### T2DM: current treatment options

The aim of T2DM treatment is to reduce permanently lower elevated blood sugar levels to a healthier level, which is the only way to prevent serious secondary diseases. Overall, T2DM treatment follows a four-level scheme. Each stage is applied for 3–6 months. If the individual hemoglobin A1c (HbA1c) target value of 6.5% to 7.5% cannot be reached during this period, the next stage of T2DM treatment begins. The basis therapy is a change in lifestyle with a change in diet, more exercise, reduction of overweight, and smoking cessation. In addition, patients should attend diabetes training. In stages 2 and 3, oral antidiabetics are used, whereas insulin therapy is the standard procedure in stage 4.^11,12^

### Lack of specialists

Diabetes patients should be encouraged by patient empowerment measures to take responsibility for promoting their own health status to implement lifestyle changes underlying the therapy and the regularly required blood glucose measurements on a long-term basis. This, however, requires professional support from family doctors and diabetologists.^13^ The current care situation shows that the German healthcare system is not sufficiently prepared for the increasing incidence of T2DM,^14^ in particular because there is a lack of diabetologists and endocrinologists (especially in rural districts), so T2DM patients may be underserved in some areas.^15,16^

### T2DM as an economic burden

The rising prevalence of T2DM has led to a growing economic burden. The total direct costs for people with DM were estimated at €7.4 billion in 2015.^17,18^ Estimates of additional costs for people with DM compared with people without DM based on German National Health Insurance accounting data from 2009, taking into account concomitant and secondary diseases, are at least €21 billion.^18–20^ In 2005, annual direct mean costs per person with diabetes amounted to €5,262, and indirect costs €5,019.^21^ Individuals with T2DM had 1.81 (95% confidence interval [CI] 1.56–2.11) times higher direct (€3,352 vs. €1,849) and 2.07 (95% CI 1.51–2.84) times higher indirect (€4,103 vs. €1,981) annual costs than those without diabetes.^22^ It is important to note that a significant proportion of diabetes-related secondary diseases and the associated healthcare costs could be avoided by early detection and prevention of T2DM.^23^

### Telemonitoring services in diabetes care

To ensure healthcare against the background of rising prevalence rates, and to reduce costs, it makes sense to supplement the treatment of T2DM patients with telemedical services. These are medical procedures intended to overcome geographical barriers, connecting users who are not in the same physical location, for example, through app-based services.^24^ Study results have indicated that telemonitoring services can reduce HbA1c levels and blood pressure in DM patients.^25^ In another study, the use of a home telehealth system was associated with better metabolic control and health-related quality of life (HRQoL).^26^ Other studies have shown that telemonitoring services can reduce HbA1c levels and improve HRQoL,^27–29^ whereas young patients (<55 years) and patients with a shorter diabetes disease history (<8.5 years) benefit the most from telemedicine.^29–31^ Physicians themselves have evaluated the use of telemedical application with DM patients largely positively: Their top three perceived benefits of telemonitoring were enhanced quality of treatment, better therapy adjustment, and reduced travel and waiting times for patients. The top three barriers were reduced personal communication, practitioner time expenditure, and (equally placed) poor financial compensation; others included data security and privacy issues.^32^ Despite the opportunities and advantages they present, telemonitoring services are not yet sufficiently utilized in the western world.^33^ The aim of this study is to evaluate telemonitoring services for DM patients in Germany and the related political and legal environment. Specifically, the relevant areas of information on the state of research in the telemedical care of diabetes patients are presented and categorized in terms of content.

## Methods

### Narrative review

A methodological narrative review was conducted. These ordinarily provide a broad overview of a specific topic. They are well suited for quickly obtaining information on the current state of research. However, the selection of articles is subjective and unsystematic, which is why such reviews should be carried out in areas where little scientific literature can be identified.^34,35^ After an initial Google search for existing published reviews (in July 2020), a PubMed search (establishing a start set with English key terms) was conducted. The same search terms were used in both cases: “Telemedicine,” “telemonitoring,” “chronic diseases,” “diabetes,” and “Germany” (filter: Title/Abstract). The selection was based on the use of interactive telemonitoring, connection or transfer, and compatibility options in German diabetes care. Owing to rapid technological progress, only articles published in and after 2015 were considered. Relevant statements from the German Diabetes Association, as well as legal requirements (e.g., of the German Federal Ministry for Health) were also taken into account; these were returned from Google searches. Because only a few articles on telemedicine care for DM patients were available, publications dealing with the possibilities of telemedicine in the case of other chronic diseases were also considered.

### Article selection and content analysis

**FIG. 1.**
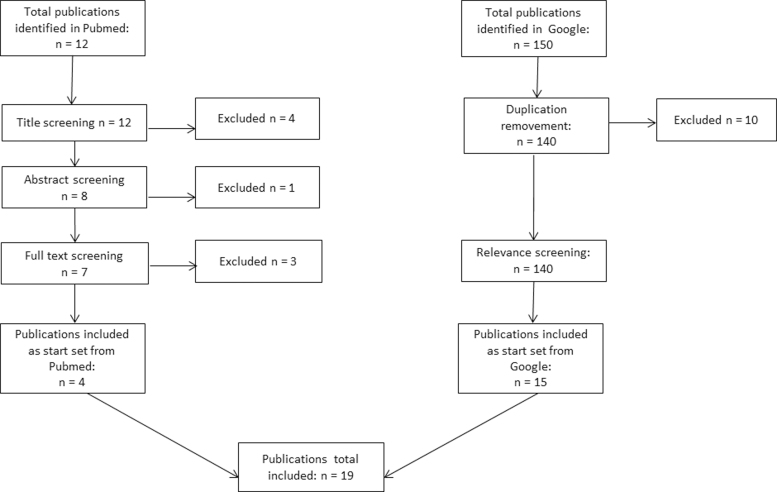
Flow diagram of publication screening and identification.

Altogether, four inclusion and exclusion criteria were each applied to the Google and PubMed searches (Table 1). Studies with a focus on DM or other chronic diseases, which reflected telemonitoring aspects of the German healthcare reality and were not older than 6 years, were included. The search was limited to 6 years because much of the legislation necessary for the implementation of telemedical services in Germany was not passed until 2015 and beyond. Only studies and reports by German authors or publications that referred directly to the German healthcare system were considered. Figure 1 shows a flow diagram of the publication screening and identification. A total of 19 publications were viewed as relevant in the screening. They were then included in the content analysis.

**Table 1.** Inclusion and Exclusion Criteria

Inclusion criteria	Exclusion criteria
- Telemonitoring treatment in DM or other chronic diseases	- Telemonitoring treatment in acute diseases
- Telemonitoring aspects in Germany	- Telemonitoring aspects in other countries
- English- or German-based publications	- Non-English- or German-based publications
- Publication date: 2015 or later	- Publication date before 2015
- Types of literature: RCTs, meta-analyses (systematic reviews), published reports of professional societies (e.g., German Diabetes Association), and German legal texts (published)	- Types of literature: unpublished texts, reports from daily newspapers and magazines that lacked scientific evidence

DM, diabetes mellitus; RCTs, randomized controlled trials.

With regard to content, all articles were assigned to three categories based on the qualitative content analysis of Mayring methodology (i.e., by generating inductive categories in accordance with explicit information in the articles).

## Results

The results were differentiated in terms of political/legal requirements, needs, and supply-related aspects so they could be located in those three dimensions. Table 2 shows the 19 studies categorized according to their content.

**Table 2.** Overview of the Selected Studies and Their Allocation to the Individual Dimensions

Publications in the dimension political/legal environment	Publications in the dimension needs for DM patients: providers' view	Publications in the dimension supply-related aspects
Deutscher Bundestag (2003)^36^ Krüger-Brand (2003)^37^ Bundesministerium für Wirtschaft und Energie (2018)^38^ Bundesärztekammer^39^ Bundesgesetzblatt (2015)^40^ Krüger-Brand: Online-Videosprechstunde (2018)^41^ Krüger-Brand: Fernbehandlung (2018)^42^ Kassenärztliche Bundesvereinigung (2020)^43^ Bundesärztekammer (2019)^44^ Bundesministerium für Gesundheit (2020)^45^	Deutsche Diabetes Gesellschaft (2018)^46^ Ickrath (2018)^47^	Waschkau et al. (2019)^48^ von Sengbusch et al. (2020)^49^ Sommer et al. (2020)^50^ Frielitz et al. (2020)^51^ Röhling et al. (2019)^52^ Köhler et al. (2019)^53^ Eder et al. (2018)^54^

### Political/legal environment

In terms of the political/legal environment, specific legislation regarding healthcare needs to be borne in mind. Since 2004, the German legislature has been working on the establishment of a telematics infrastructure (TI) and the introduction of an electronic health card based on the Healthcare Modernization Act (Gesundheitsmodernisierungsgesetz).^36,37^ Telematics includes telecommunication and informatics. It describes the networking of different IT systems and the possible linkage of information from different sources. The TI should connect all market players in the healthcare system to ensure secure transfer of information; this is fundamental for telemedicine solutions. The TI also defines who is authorized to access data. To this end, the gematik are integrating certified components into the systems. Extensive technological structures and applications for telemedicine have not yet been implemented because of the gaps in nationwide internet broadband supply.^38,39^ The E-Health Law (Gesetz für sichere digitale Kommunikation und Anwendungen im Gesundheitswesen sowie zur Änderung weiterer Gesetze), which was passed in 2016, aims to establish a telemedicine framework that ensures data security, so the patient's competence to make decisions about his or her personal and health data is enhanced. All relevant data are saved in one place and are distributed to multidisciplinary healthcare providers.^38–40^ In 2017, a new resolution was passed, which stated that video consulting hours (in German: Online Video-Sprechstunde) could be invoiced. The agreement was further extended so that after basic invoicing is permitted after an initial physical face-to-face consultation, and no direct contact is then required.^41^ Furthermore, prohibition of remote treatment (in German: Fernbehandlungsverbot) was removed from the medical code of conduct (Musterberufsordnung) in 2018.^42^ In 2020, video consultations were temporarily massively expanded in the context of the coronavirus disease 2019 (COVID-19) pandemic.^43^ In 2019, the Digital Provision Law (in German: Digital-Versorgungs-Gesetz) and the Digital Health Applications Regulation (in German: Digitale Gesundheitsanwendungen-Verordnung) were passed to ensure that digital applications marketed as medical devices meet high quality standards.^44,45^ The necessary legal requirements for the standard implementation of telemedical procedures have still not been completed in Germany, and a number of hurdles in technical implementation and data protection remain.

### DM patients' needs: the providers' view

With regard to content analysis, certain items of inductive information generate a dimension of needs for DM patients, but only from a professional point of view, since needs in terms of the patients' perspective have not yet been systematically ascertained in Germany, which is why the German Diabetes Society's (Deutsche Diabetes Gesellschaft [DDG]) recommendations have been used. Against the background of the growing epidemiological importance of DM and gaps in healthcare, the DDG promotes the massive expansion of telemedical services. It has published a code of conduct with seven concrete areas of action^46^:

1. Data and information protection: The DDG demands the necessary requirements for the self-determination of patients in relation to their data. It demands a balance of restrictions in data security to allow patients to participate in research-based diabetes (technology) development.
2. Data security: In addition to the rules concerning the European Union's basic regulation of data protection, the DDG requires that IT systems should be connected. This connection does not currently exist. The DDG expects improvements in internet security to shield patient data from the government and industry and to secure the data structures of medical offices.
3. Interoperability: The DDG welcomes the health law (and the creation of a TI), but criticizes its inadequate implementation. The DDG demands that companies working in the field of diabetology system development are committed to the integration of technical standards, so the digitization of patient care allows for trans-sectoral collaboration.
4. Digital treatment standards: The DDG plans to establish a German electronic health ID card for diabetes (in German: elektronische Diabetesakte) to leverage structured, guideline-orientated, and patient-centered diabetes treatment and to build a register for diabetes data for research.
5. Effective physician−patient relationships (in German: Sprechende Medizin): In terms of personalized medicine, the DDG requires more focus on therapy and adequate compensation, which are key elements in diabetes treatment, to analyze behavior and provide motivation.
6. Training: The DDG is committed to working on training in data protection, security, and technology.
7. Algorithms and transparency: The DDG demands a national framework for an algorithm of diabetes health data, so that the interpretation serves the well-being of the patient.^46,47^

### Supply-related aspects

Only four studies focus on the individual care aspects of telemedical care of DM patients, although Waschkau et al. have pointed that in 2019, 12 projects were being conducted and evaluated in Germany.^48^ Individual studies investigate parental expectations before and after a 12-month experience with video consultations combined with regular outpatient care for children with T1DM,^49^ and the preferences of people with T2DM for telemedical lifestyle programs in Germany.^50^ Other studies examine whether monthly telemedical consultations, in addition to regular care, will improve glycemic control and psychosocial outcomes in children with T1DM^51^ and the clinical impact of an interprofessional and telemedicine-based diabetes management system.^52^ Few German studies have investigated the application of telemedicine to other chronic illnesses. One presents the technical and organizational basics of telemedical methods in outpatient heart failure care,^53^ whereas another evaluates the prehospital telemedical emergency management of severely injured trauma patients.^54^

## Discussion

### Main results

Telemonitoring measures for chronic diseases in general and for DM in particular have yet to be widely implemented in Germany. Only a few projects evaluate the individual dimension, whereas those that focus on effectiveness are even more scarce.^48,55^ This is partly due to the fact that not all legal hurdles for the application of telemedicine have been removed; problems with technical implementation and data protection still exist; and financing mechanisms have not yet been completely clarified. The demands of the DDG encapsulate the essential needs of DM patients with regard to the expansion of telemedicine, but it may be assumed that, in light of the aforementioned issues, these will not be implemented for several years.^56^ Studies are underway on the clinical effects of such services, the preferences and expectations of patients, and whether glycemic control can be improved.^49–52^

### Limitations

The aim of this study was to evaluate telemonitoring services for DM patients in Germany and the political/legal environment. Since only a few studies exist, a narrative review was conducted rather than a systematic search for PRISMA criteria. This explains the study's limitations. Narrative reviews are considered to be susceptible to subjective influences, because the literature is not selected according to strictly objective criteria and the results are, therefore, bias sensitive. In this case, since only German and English publications from the past 6 years were included, it is not clear whether all relevant studies were considered. In contrast, the exclusion criteria were appropriate for the German context.

### Effectiveness of telemedical care of DM

Although telemedical applications for chronic diseases are considered to offer great potential, the state of research is extremely deficient. Although there are indications that telemonitoring services can reduce HbA1c levels and blood pressure in DM patients^25^ and can improve HRQoL,^26–29^ other studies suggest that there is no clear evidence that teleconsultation can improve the care of DM-associated chronic wounds.^57^ Nevertheless, there is a high level of agreement among patients with chronic diseases (especially in the context of the COVID-19 pandemic) of the benefits; they just have to be realized.^58,59^ Against this background, telemedical measures must first be implemented and critically evaluated before they can be transferred to standard care. The legislator is called upon to remove the existing hurdles as soon as possible. This will also meet the requirements of the World Health Organization for improvements in the care of all patients with chronic diseases, regardless of social status.^60^

## Conclusion

This study can make an essential contribution to future research processes and to a theoretical framework for innovation research. Based on the deficiencies and research gaps described earlier, a number of recommendations can be made. There is a need to set up a more interactive structure, to expand the present technical infrastructure, and to close legal gaps. More research that focuses on clinical effectiveness is necessary. Pilot projects incorporating structured evaluations with existing telemedicine centers could be implemented. Co-operation between service providers, physicians, insurance companies, and politicians to improve the telemedical care of DM patients should be encouraged. Given the high prevalence of DM and the resulting high costs for the healthcare system, it remains an open question as to why telemedicine has not received greater attention. The current pandemic should accelerate the use of appropriate telemedical services in the area of diabetes care. As has been mentioned, legal barriers are currently being addressed by legislation, whereas the DDG is pressing for the introduction of a range of measures. The challenge now is to implement systems and to assess needs from the patient's perspective, so that care is improved and made more efficient.

## Data Availability

The data sets used and/or analyzed during this study are available from the corresponding author upon reasonable request.

## Abbreviations Used

CI: confidence interval
COVID-19: coronavirus disease 2019
DDG: Deutsche Diabetes Gesellschaft
DM: diabetes mellitus
HbA1c: hemoglobin A1c
HRQoL: health-related quality of life
T1DM: type 1 diabetes mellitus
T2DM: type 2 diabetes mellitus
T3DM: type 3 diabetes mellitus
TI: telematics infrastructure

## References

[1] Kharroubi AT, Darwish HM. Diabetes mellitus: the epidemic of the century. World J Diabetes 2015;6:850–867. 26131326 10.4239/wjd.v6.i6.850 PMC4478580

[2] American Diabetes Association. Diagnosis and classification of diabetes mellitus [published correction appears in Diabetes Care 2010;33(4):e57]. Diabetes Care 2010;33(Suppl. 1):S62–S69. 20042775 10.2337/dc10-S062 PMC2797383

[3] Fajans SS, Bell GI. MODY: history, genetics, pathophysiology, and clinical decision making. Diabetes Care 2011;34:1878–1884. 21788644 10.2337/dc11-0035 PMC3142024

[4] Herder C, Roden M. Genetics of type 2 diabetes: pathophysiologic and clinical relevance. Eur J Clin Invest 2011;41:679–692. 21198561 10.1111/j.1365-2362.2010.02454.x

[5] International Diabetes Federation. IDF Diabetes Atlas, 9th ed. 2019. Brussels, Belgium: IDF; 2019. Available at https://www.diabetesatlas.org.

[6] Jacobs E, Rathmann W. Epidemiology of Diabetes in Germany (in German). Diabetol Stoffwechsel 2017;12:437–446.

[7] Tamayo T, Brinks R, Hoyer A, et al. The prevalence and incidence of diabetes in Germany. An analysis of statutory health insurance data on 65 million individuals from the years 2009 and 2010. Dtsch Arztebl Int 2016;113:177–182. 10.3238/arztebl.2016.0177 PMC4850517 27118665

[8] Heidemann C, Scheidt-Nave C. Prevalence, incidence and mortality of diabetes mellitus in adults in Germany—a review in the framework of the Diabetes Surveillance. J Health Monitor 2017;2:98–121. 10.17886/RKI-GBE-2017-062 PMC10165910 37168946

[9] Tamayo T, Rosenbauer J, Wild SH, et al. Diabetes in Europe: an update. Diabetes Res Clin Pract 2014;103:206–217. 24300019 10.1016/j.diabres.2013.11.007

[10] Rathmann W, Haastert B, Icks A, et al. The diabetes epidemic in the elderly population in Western Europe: data from population-based studies. Gesundheitswesen 2005;67(Suppl. 1):S110–S114. 16032527 10.1055/s-2005-858227

[11] Marín-Peñalver JJ, Martín-Timón I, Sevillano-Collantes C, Del Cañizo-Gómez FJ. Update on the treatment of type 2 diabetes mellitus. World J Diabetes 2016;7:354–395. 27660695 10.4239/wjd.v7.i17.354 PMC5027002

[12] Pfeiffer AFH, Klein HH. The treatment of type 2 diabetes. Dtsch Arztebl Int 2014;111:69–82. 24612534 10.3238/arztebl.2014.0069 PMC3952010

[13] Gómez-Velasco DV, Almeda-Valdes P, Martagón AJ, et al. Empowerment of patients with type 2 diabetes: current perspectives. Diabetes Metab Syndr Obes 2019;12:1311–1321. 31496769 10.2147/DMSO.S174910 PMC6689555

[14] Hasseler MK, von der Heide M, Indefrey S. Resources for and barriers to effective diabetes care management—experiences and perspectives of people with type 2 diabetes. J Public Health 2011;19:65–71.

[15] Laxy M, Knoll G, Schunk M, et al. Quality of diabetes care in Germany improved from 2000 to 2007 to 2014, but improvements diminished since 2007. Evidence from the population-based KORA studies. PLoS One 11:e0164704. 10.1371/journal.pone.0164704 PMC5066975 27749939

[16] Deutsches Ärzteblatt. Diabetology: Lack of specialists, increasing demand (in German). November 22, 2019. Available at https://www.aerzteblatt.de/nachrichten/107637/Diabetologie-Fehlender-Nachwuchs-steigender-Bedarf Accessed September 28, 2020.

[17] Statistisches Bundesamt (Destatis). Medical expenses for diabetes mellitus (in German). 2019. Available at https://www-genesis.destatis.de/genesis/online/logon?sequenz=tabelleEr-gebnis&selectionname=23631-0003&sachmerkmal=IC-D10Y&sachschluessel=ICD10-E10-E14&transponie-ren=true Accessed September 28, 2020.

[18] Robert-Koch-Institut. Diabetes in Germany. Report of the National Diabetes-Surveillance (in German), 2019. Available at https://diabsurv.rki.de/SharedDocs/downloads/DE/DiabSurv/diabetesbericht2019.pdf?__blob=publicationFile&v=12 Accessed September 28, 2020.

[19] Jacobs E, Hoyer A, Brinks R, et al. Healthcare costs of type 2 diabetes in Germany. Diabet Med 2017;34:855–861. 28199029 10.1111/dme.13336

[20] Köster I, Schubert I, Huppertz E. Update of the KoDiM study: Costs of diabetes mellitus 2000–2009 (in German). Dtsch Med Wochenschr 2012;137:1013–1016. 22549261 10.1055/s-0032-1304891

[21] Köster I, von Ferber L, Ihle P, et al. The cost burden of diabetes mellitus: the evidence from Germany—the CoDiM study. Diabetologia 2006;49:1498–1504. 16752168 10.1007/s00125-006-0277-5

[22] Ulrich S, Holle R, Wacker M, et al. Cost burden of type 2 diabetes in Germany: results from the population-based KORA studies. BMJ Open 2016;6:e012527. 10.1136/bmjopen-2016-012527 PMC5129071 27872118

[23] Schaufler TM. Economic benefits of early medical diagnostics: Economic evaluation using the example of a screening after diabetes mellitus type 2 (health management and medical economics) (in German). Hamburg: Verlag Dr. Kovac, 2007.

[24] Gilman M, Stensland J. Telehealth and Medicare: payment policy, current use, and prospects for growth. Med Medicaid Res Rev 2013;3:E1–E17. 10.5600/mmrr.003.04.a04 PMC4011650 24834368

[25] Shane-McWhorter L, Lenert L, Petersen M, et al. The Utah Remote Monitoring Project: improving health care one patient at a time. Diabetes Technol Ther 2014;16:653–660. 24991923 10.1089/dia.2014.0045 PMC4183896

[26] Nicolucci A, Cercone S, Chiriatti A, Muscas F, Gensini G. A randomized trial on home telemonitoring for the management of metabolic and cardiovascular risk in patients with type 2 diabetes. Diabetes Technol Ther 2015;17:563–570. 26154338 10.1089/dia.2014.0355

[27] Katalenich B, Shi L, Liu S, et al. Evaluation of a remote monitoring system for diabetes control. Clin Ther 2015;37:1216–1225. 25869625 10.1016/j.clinthera.2015.03.022 PMC4496944

[28] Wild SH, Hanley J, Lewis SC, et al. Supported telemonitoring and glycemic control in people with type 2 diabetes: the telescot diabetes pragmatic multicenter randomized controlled trial [published correction appears in PLoS Med 2016 Oct 19;13(10):e1002163]. PLoS Med 2016;13:e1002098. 27458809 10.1371/journal.pmed.1002098 PMC4961438

[29] Lee SWH, Chan CKY, Chua SS, Chaiyakunapruk N. Comparative effectiveness of telemedicine strategies on type 2 diabetes management: a systematic review and network meta-analysis. Sci Rep 2017;7:12680. 28978949 10.1038/s41598-017-12987-z PMC5627243

[30] Wu IXY, Kee JCY, Threapleton DE, et al. Effectiveness of smartphone technologies on glycaemic control in patients with type 2 diabetes: systematic review with meta-analysis of 17 trials. Obes Rev 2018;19:825–838. 29345109 10.1111/obr.12669

[31] Zhai YK, Zhu WJ, Cai YL, Sun DX, Zhao J. Clinical- and cost-effectiveness of telemedicine in type 2 diabetes mellitus: a systematic review and meta-analysis. Medicine (Baltimore) 2014;93:e312. 25526482 10.1097/MD.0000000000000312 PMC4603080

[32] Muigg D, Kastner P, Duftschmid G, Modre-Osprian R, Haluza D. Readiness to use telemonitoring in diabetes care: a cross-sectional study among Austrian practitioners. BMC Med Inform Decis Mak 2019;19:26. 30696444 10.1186/s12911-019-0746-7 PMC6352347

[33] Andrès E, Meyer L, Zulfiqar AA, et al. Telemonitoring in diabetes: evolution of concepts and technologies, with a focus on results of the more recent studies. J Med Life 2019;12:203–214. 31666818 10.25122/jml-2019-0006 PMC6814890

[34] Montori VM, Swiontkowski MF, Cook DJ. Methodologic issues in systematic reviews and meta-analyses. Clin Orthop Relat Res 2003;413:43–54. 10.1097/01.blo.0000079322.41006.5b 12897595

[35] Ressing M, Blettner M, Klug SJ. Systematic reviews and meta-analyzes. Part 6 of the series on the evaluation of scientific publications (in German). Dtsch Arztebl Int 2009;106:456–463. 10.3238/arztebl.2009.0456 PMC2719096 19652768

[36] Deutscher Bundestag. Draft law of the German parliamentary groups SPD, CDU/CSU and BÜNDIS 90/Die Grünen. Draft law on modernization of the statutory health insurance (in German) (GKV-Modernisierungsgesetz—GMG). Drucksache 15/1525. Available at http://dipbt.bundestag.de/doc/btd/15/015/1501525.pdf Accessed October 6, 2020.

[37] Krüger-Brand HE. Telematics framework architecture: Concepts, scenarios, friction (in German). Dtsch Arztebl 2003;100:A-1047.

[38] Bundesministerium für Wirtschaft und Energie (2018): Annual report of the federal government on the state of German unity (in German). Berlin, 2018. Available at https://www.bmwi.de/Redaktion/DE/Publikationen/Neue-Laender/jahres-bericht-zum-stand-der-deutschen-einheit-2018.pdf?__blob=publication-File&v=14 Accessed July 15, 2020.

[39] Bundesärztekammer. E-Health-Law—new applications for physicians and insured people (in German). Available at https://www.bundesaerztekammer.de/aerzte/telematiktelemedizin/earztausweis/e-health-gesetz Accessed October 6, 2020.

[40] Bundesgesetzblatt. Law for secure digital communication and healthcare applications as well as for modifying other laws (in German). Bundesgesetzblatt 2015;I:2408–2423.

[41] Krüger-Brand HE. Online video consultation: Proven tool (in German). Dtsch Arztebl 2018;115:A-212/B-184/C-184.

[42] Krüger-Brand HE. Remote treatment: Open the way for telemedicine (in German). Dtsch Arztebl 2018;115:A-965/B-813/C-813.

[43] Kassenärztliche Bundesvereinigung. Coronavirus: Video consultation hours possible without restrictions (in German). Available at https://www.kbv.de/html/1150_44943.php Accessed October 6, 2020.

[44] Bundesärztekammer. Statement of the German Medical Association on the draft law for a better medical treatment as a result of digitization and innovation (in German) (Digitale Versorgung-Gesetz—DVG). 2019. Available at https://www.bundesaerztekammer.de/fileadmin/user_upload/downloads/pdf-Ordner/Stellungnahmen/DVG-RefE.pdf Accessed October 6, 2020.

[45] Bundesministerium für Gesundheit. DiGAV, Digitale-Gesundheitsanwendungen-Verordnung 2020, Regulation on the procedure and requirements for the proof of reimbursement of digital health applications within the statutory health insurance (in German) legal draft. Available at https://www.bundesgesundheitsministerium.de/fileadmin/Dateien/3_Downloads/Gesetze_und_Verordnungen/GuV/D/DiGAV_RefE.pdf Accessed October 6, 2020.

[46] Deutscher Diabetes Gesellschaft. Framework document for a code of Conduct for Digital Health of the German Diabetes Society (DDG) for digital transformation. Berlin, 2018. Available at https://www.deutsche-diabetes-gesellschaft.de/fileadmin/user_upload/06_Gesundheitspolitik/03_Veroeffentlichungen/01_Code_of_Conduct_Digital_Health/Code_of_Conduct_der_DDG_Digital_Health_19092017.pdf Accessed October 6, 2020.

[47] Ickrath M. Importance of digitalization for the DDG as a professional society. Design requirements and orientation guide. Der Diabetologe 2018;14:449–454.

[48] Waschkau A, Uebel T, Steinhäuser J. Diabetestherapie 2.0—Telemedizin [Diabetes treatment 2.0: telemedicine]. Internist (Berl) 2019;60:917–924. 31346638 10.1007/s00108-019-0650-3

[49] von Sengbusch S, Doerdelmann J, Lemke S, et al. Parental expectations before and after 12-month experience with video consultations combined with regular outpatient care for children with type 1 diabetes: a qualitative study. Diabet Med 2020;24:e14410. 10.1111/dme.14410 32969088

[50] Sommer J, Dyczmons J, Grobosch S, et al. Preferences of people with type 2 diabetes for telemedical lifestyle programmes in Germany: protocol of a discrete choice experiment. BMJ Open 2020;10:e036995. 10.1136/bmjopen-2020-036995 PMC7482475 32907900

[51] Frielitz FS, Müller-Godeffroy E, Hübner J, et al. Monthly video-consultation for children with type 1 diabetes using a continuous glucose monitoring system: design of ViDiKi, a multimethod intervention study to evaluate the benefit of telemedicine. J Diabetes Sci Technol 2020;14:105–111. 31315446 10.1177/1932296819861991 PMC7189148

[52] Röhling M, Redaélli M, Simic D, et al. TeDia.—A telemedicine-based treatment model for Inpatient and interprofessional diabetes care. Diabetes Metab Syndr Obes 2019;12:2479–2487. 31819573 10.2147/DMSO.S229933 PMC6890178

[53] Köhler F, Prescher S, Köhler K. Telemedizin bei Herzinsuffizienz [Telemedicine in heart failure]. Internist (Berl) 2019;60:331–338. 30820589 10.1007/s00108-019-0570-2

[54] Eder PA, Reime B, Wurmb T, et al. Prehospital telemedical emergency management of severely injured trauma patients. Methods Inf Med 2018;57:231–242. 30875702 10.1055/s-0039-1681089

[55] Allner R, Wilfling D, Kidholm K, Steinhäuser J. Telemedicine projects in rural area of Germany. A systematic evaluation using the “Model for the evaluation of telemedical applications” Regulation on the procedure and requirements for the proof of reimbursement of digital health applications within the statutory health insurance (in German). Z Evid Fortbild Qual Gesundhwes 2019;141–142:89–95.

[56] Zippel-Schultz B, Schultz C, Helms TM. Current status and future of telemonitoring: scenarios for telemedical care in 2025. Herzschr Elektrophys 2017;28:245–256. 10.1007/s00399-017-0520-4 28849391

[57] Hrynyschyn R, Dockweiler C, Iltner J, Hornberg C. Teleconsultation for chronic vascular disease and diabetic chronic wounds: A systematic review of health related and economic implications Regulation on the procedure and requirements for the proof of reimbursement of digital health applications within the statutory health insurance (in German). Hautarzt 2020;71:114–123. 31659390 10.1007/s00105-019-04498-x

[58] Thielscher C, Doarn CR. Long-term future of telemedicine in Germany: the patient's, physician's, and payer's perspective. Telemed J E Health 2008;14:701–706. 18817500 10.1089/tmj.2007.0104

[59] Boehm K, Ziewer, S, Brandt MA, et al. Telemedicine online visits in urology during the COVID-19 pandemic—potential, risk factors, and patients' perspective. Eur Urol 2020;78:16–20. 32362498 10.1016/j.eururo.2020.04.055 PMC7183955

[60] World Health Organization (WHO). Telemedicine: opportunities and developments in Member States: report on the second global survey on eHealth 2009, Global Observatory for eHealth Series 2. WHO Library Cataloguing-in-Publication Data, 2020. Available at https://apps.who.int/iris/bitstream/handle/10665/44497/9789241564144_eng.pdf?sequence=1&isAllowed=y Accessed October 6, 2020.

